# Expression of a protease-resistant insulin-like growth factor-binding protein-4 inhibits tumour growth in a murine model of breast cancer

**DOI:** 10.1038/sj.bjc.6605141

**Published:** 2009-06-16

**Authors:** A J Ryan, S Napoletano, P A Fitzpatrick, C A Currid, N C O'Sullivan, J H Harmey

**Affiliations:** 1 Molecular and Cellular Therapeutics, Royal College of Surgeons in Ireland, 123 St Stephens Green, Dublin 2, Ireland

**Keywords:** insulin-like growth factor, insulin-like growth factor-binding protein-4, pregnancy-associated plasma protein A, angiogenesis, breast cancer

## Abstract

**Background:**

Insulin-like growth factor 1 (IGF1) promotes breast cancer and disease progression. Bioavailability of IGF1 is modulated by IGF-binding proteins (IGFBPs). IGFBP4 inhibits IGF1 activity but cleavage by pregnancy-associated plasma protein-A (PAPP-A) protease releases active IGF1.

**Methods:**

Expression of IGF pathway components and PAPP-A was assessed by western blot or RT–PCR. IGFBP4 (dBP4) resistant to PAPP-A cleavage, but retaining IGF-binding capacity, was used to block IGF activity *in vivo*. 4T1.2 mouse mammary adenocarcinoma cells transfected with empty vector, vector expressing wild-type IGFBP4 or vector expressing dBP4 were implanted in the mammary fat pad of BALB/c mice and tumour growth was assessed. Tumour angiogenesis and endothelial cell apoptosis were assessed by immunohistochemistry.

**Results:**

4T1.2 cells expressed the IGF1R receptor and IGFBP4. PAPP-A was expressed within mammary tumours but not by 4T1.2 cells. Proliferation and vascular endothelial growth factor (VEGF) production by 4T1.2 cells was increased by IGF1(E3R) (recombinant IGF1 resistant to binding by IGFBPs) but not by wild-type IGF1. IGF1-stimulated microvascular endothelial cell proliferation was blocked by recombinant IGFBP4. 4T1.2 tumours expressing dBP4 grew significantly more slowly than controls or tumours expressing wild-type IGFBP4. Inhibition of tumour growth by dBP4 was accompanied by the increased endothelial cell apoptosis.

**Conclusion:**

Protease-resistant IGFBP4 blocks IGF activity, tumour growth and angiogenesis .

Insulin-like growth factors (IGFs) are small potent mitogenic proteins and there is significant evidence that IGF1 promotes breast cancer and plays a role in disease progression (Hankinson *et al*, 1998). The type I insulin-like growth factor receptor (IGF1R) is overexpressed by many breast cancer cell lines and high levels of IGF1 are associated with poor prognosis in breast cancer (Huynh *et al*, 1993; Peyrat *et al*, 1993). IGF1 (or 2) binding results in autophosphorylation of IGF1R and activation of PI3K (phosphatidylinositol-3-kinase), Akt and MAPK (mitogen-activated protein kinase) (LeRoith *et al*, 1995; Saltiel and Kahn, 2001). The insulin-like growth factor-binding proteins (IGFBPs) modulate the biological activity of IGF, either inhibiting or potentiating its effects. At least six IGFBPs have been identified to date. Proteolysis of the IGFBPs plays a vital role in regulating IGF activity (Fowlkes *et al*, 1997) releasing biologically active IGF (Clemmons *et al*, 1998).

IGFBP4 inhibits the activity of IGF1 and does not enhance the actions of IGF1 under any conditions (Jones and Clemmons, 1995). IGFBP4 is produced mainly in the liver but many other tissues, especially neoplastic tissue, produce it (Korc-Grodzicki *et al*, 1993; Lewinski *et al*, 2004).

IGFBP4 protease activity has been described in a variety of cell types including placental tissue, fibroblasts and osteoblasts (Conover *et al*, 1993; Durham *et al*, 1995). The IGFBP4 protease is a metalloprotease, which cleaves IGFBP4 into 18 and 14 kDa fragments and its activity is IGF-dependent (Conover *et al*, 1995). It was later identified as pregnancy-associated plasma protein-A (PAPP-A) (Lawrence *et al*, 1999) with a cleavage site at Met^135^-Lys^136^ in the mature human IGFBP4 protein (Conover *et al*, 1995; Chelius *et al*, 2000). Although IGFBP4 inhibits the actions of IGF1, proteolytic cleavage by PAPP-A *in vivo* releases biologically active IGF from IGFBP4. In addition to PAPP-A, prostatic kallikreins, hK2 and hK3 (prostate specific antigen), can also cleave IGFBP4 (Rehault *et al*, 2001).

We established that 4T1.2 mouse mammary adenocarcinoma cells expressed IGF1R and were responsive to IGF1. We anticipated that wild-type IGFBP4 would be unable to block IGF1 activity in 4T1.2 mammary tumours *in vivo* because of the presence of active PAPP-A in mammary tissue. We hypothesised that a protease-resistant IGFBP4, however, would inhibit 4T1.2 tumour growth by binding IGF1 rendering it biologically inactive even in the presence of PAPP-A. In the orthotopic 4T1.2 breast cancer model, we demonstrate that the expression of a protease-resistant IGFBP4, where the PAPP-A cleavage site has been mutated without altering its IGF-binding capacity, inhibited 4T1.2 tumour growth. PAPP-A sensitive wild-type IGFBP4 had no significant effect on tumour growth. Inhibition of tumour growth was accompanied by increased endothelial cell apoptosis within the tumours.

## Materials and methods

### Cell culture

4T1.2 cells, murine mammary adenocarcinoma cells that preferentially metastasise to bone, were a gift from Robin Anderson (Peter MacCallum Cancer Centre, Australia.) (Lelekakis *et al*, 1999). 4T1.2 cells were maintained in Roswell Park Memorial Institute (RPMI) 1640 medium, supplemented with 10% (v/v) fetal calf serum (FCS), 100 U ml^−1^ penicillin, and 100 *μ*g ml^−1^ streptomycin sulphate. MCF7 human breast adenocarcinoma cells were maintained in Eagles Minimum Essential Medium supplemented with 10% (v/v) FCS. Human embryonic kidney (HEK) 293 cells were maintained in high-glucose Dulbecco's Modified Eagle's Medium (DMEM) supplemented with 10% (v/v) FCS. MC3T3 osteoblasts were differentiated for 7 days in *α*MEM containing 10% (v/v) FCS supplemented with 10 mM *β*-glycerol phosphate and 50 *μ*g ml^−1^ ascorbic acid. Primary dNeo-Neonatal human dermal microvascular endothelial cells (MVECs) were purchased from Lonza (Lonza Workingham Ltd, Workingham, UK) and maintained in complete EGM-2MV Microvascular Endothelial Cell Growth Medium-2 (Lonza Workingham Ltd, Workingham, UK) (containing gentamicin, 5% (v/v) FCS, hEGF, hydrocortisone, VEGF, hFGF-B, R^3^-IGF-1 and ascorbic acid). All cell lines were cultured at 37°C in an atmosphere of 5% CO_2_ in air.

### Effect of IGF1 on 4T1.2 cell proliferation

10^3^ 4T1.2 cells per well were plated in triplicate in 96-well plates, allowed to adhere overnight, then treated with recombinant mouse IGF1 (R&D Systems, Abingdon, UK) or IGF1 (E3R) (Upstate, Cell Signaling Solutions, Lake Placid, NY, USA) for 48 h at 0–100 ng ml^−1^. Following treatment, cell proliferation was assessed using a colourimetric cell proliferation BrdU ELISA Kit (Roche Diagnostics, Burgess Hill, UK). Proliferation was expressed relative to untreated controls, which were taken as 100%.

### Effect of IGF1 on MVEC proliferation

1500 MVEC cells per well were plated in duplicate in 96-well plates, allowed to adhere overnight, then treated for 96 h in complete EGM-2MV medium with 50 ng ml^−1^ recombinant human IGF-1 alone or in combination with 200 ng ml^−1^ recombinant human IGFBP4, or with 10 ng ml^−1^ recombinant human VEGF alone (IGF1, IGFBP4 and VEGF from R&D Systems). Following treatment, 10 *μ*l of 5 mg ml^−1^ MTT reagent (3-[4,5-Dimethyl-2-thiazolyl]-2,5-diphenyl-2H-tetrazolium bromide, Sigma-Aldrich, St Louis, MO, USA) was added to each well for 4 h at 37°C. Culture medium was removed, MTT was solubilised in 100 *μ*l dimethyl sulphoxide (Sigma-Aldrich) and optical density at 570 nm was recorded. Proliferation was expressed relative to untreated controls, which were taken as 100%.

### Effect of IGF1 on VEGF expression

10^3^ cells per well were plated in triplicate in 96-well plates, allowed to adhere overnight, then treated with IGF1 (R&D Systems) or IGF1 (E3R) (Upstate, Cell Signaling Solutions) in serum-free medium for 48 h at 0–100 ng ml^−1^. Culture supernatants were collected and VEGF_165_ expression was assayed by ELISA (R&D Systems). Total cell protein was measured by BCA assay (Pierce, Rockford, IL, USA). VEGF_165_ was expressed as pg mg^−1^ cell protein.

### Western blot analysis

A total of 400 mg of normal mammary fat pad tissue and 4T1.2 mammary tumours was finely chopped on ice in 500 *μ*l of RIPA buffer (25 mM Tris-HCl, pH 7.4, 150 mM NaCl, 1% (v/v) NP-40, 1% (w/v) SDS, 2 mM EDTA, 1/100 dilution protease inhibitor cocktail (Sigma-Aldrich)), placed on ice for 30 min, disrupted three times (10 s each) at 12 000 r.p.m. with a Polytron homogeniser (PT1600E, Kinematica, Switzerland) and incubated on ice for a further 30 min. Homogenate was cleared by centrifuging at 12 000 **g** for 1 h at 4°C.

Conditioned medium was concentrated using 3 kDa Amicon centrifugal filters (Millipore, Billerica, MA, USA). For cell lysates, cells were washed twice with PBS and lysed in RIPA buffer. Total protein was quantified using the BCA assay (Pierce). A total of 25 *μ*g of protein was fractionated by 12% SDS–PAGE or using 4–20% Precise precast gels (Pierce) and transferred to nitro-cellulose membranes. Membranes were blocked in TBST (10 mM Tris-HCl, pH 7.4, 100 mM NaCl, 0.1% (v/v) Tween-20) containing 5% (w/v) non-fat powdered milk for 1 h and incubated overnight at 4°C with either 1 : 4000 rabbit anti-human IGFBP4 (Upstate) in TBST/5% (w/v) non-fat powdered milk or 1 : 1000 rabbit anti-human IGFIR*β* (Cell Signaling Technology Inc., Danvers, MA, USA) in TBST/5% (w/v) BSA (bovine serum albumin). Both antibodies also recognise the mouse protein. Membranes were washed six times with TBST, incubated with 1 : 2000 horseradish peroxidase conjugated anti-rabbit antibody (DAKO, Glostrup, Denmark) in TBST/5% (w/v) non-fat powdered milk, for 1 h, then washed six times with TBST. Antibody complexes were detected using Supersignal West Pico Chemiluminescent Substrate (Pierce).

### PAPP-A RT–PCR

Total RNA was isolated using TRIzol reagent (Invitrogen, Paisley, UK) according to the manufacturer's instructions. Briefly, samples were homogenized in TRIzol reagent, RNA was separated using chloroform and precipitated with isopropanol. RNA pellet was then washed with ethanol and dissolved in RNase-free water. SuperScript III First-Strand Synthesis System for RT–PCR (Invitrogen, Paisley, UK) was used to generate cDNA for PCR. PAPP-A cDNA was amplified using 50 pM forward (5′-CACTTGGGCGGTATTGTCTT-3′) and reverse (5′-TGGGTTGGTATCATTGCAGA-3′) primers, which produce a 198 bp product from mouse PAPP-A. A total of 40 cycles of 94°C for 45 s, 56°C for 45 s, 72°C for 45 s were carried out using AmpliTaq polymerase (Applied Biosystems, Foster City, CA, USA). *β*-Actin was amplified using mouse *β*-actin-specific primers (Stratagene, La Jolla, CA, USA) by 35 cycles of 94°C for 45 s, 60°C for 45 s, 72°C for 45 s.

### IGFBP4 cloning

Wild-type rat IGFBP4 (BP4) and protease-resistant rat IGFBP4 (dBP4) clones in pBluescript SK+ – pW12 and pSMP-8, respectively – were a gift from Dr James Fagin, (Division of Endocrinology, University of Cincinnati College of Medicine and the Children's Hospital Medical Centre, Cincinnati, OH, USA). In dBP4, the PAPP-A cleavage site of IGFBP4, KHMAKVRDRSKMK, was mutated to AAMAAVADASAMA, preventing proteolytic cleavage without affecting IGF-binding capacity (Zhang *et al*, 2002). BP-4 and dBP-4 were excised with *Eco*RI, ligated into pCMVScript and verified by sequencing. 4T1.2 cells were transfected with pCMV, pCMV-BP4 or pCMV-dBP4 using FuGENE 6 (Roche Diagnostics).

### PAPP-A digestion

1.5 × 10^5^ HEK293 cells per well were plated in six-well plates, allowed to adhere overnight and transfected with pCMV, pCMV-BP4 or pCMV-dBP4 using Effectene (Qiagen, Sussex, UK). Following transfection, cells were maintained in serum-free medium for 48 h. Conditioned medium was concentrated using 3 kDa Amicon centrifugal filters (Millipore) and 25 *μ*g total protein in 20 *μ*l was incubated with IGF2 (10 *μ*g ml^−1^) (R&D Systems) and rhPAPP-A (500 ng ml^−1^) for 24 h at 37°C. rhPAPP-A was a gift from Dr Claus Oxvig, University of Aarhus as described (Overgaard *et al*, 2000). Following digestion, IGFBP4 cleavage was analysed by western blot.

### *In vivo* model

Animals were housed in a licensed biomedical facility (RCSI, Beaumont Hospital) and had *ad libitum* access to animal chow and water. All procedures were subjected to institutional ethics review, carried out under animal licence guidelines of the Department of Health and Children, Ireland and in accordance with the UK Co-ordinating Committee on Cancer Research (UKCCCR) Guidelines for the Welfare of Animals in Experimental Neoplasia (1998). 5 × 10^4^ 4T1.2 cells transfected with pCMV, pCMV-BP4 or pCMV-dBP4 were implanted into the mammary fat pad of 12-week-old female BALB/c mice. Tumours were measured with calipers on alternate days and the mean tumour diameter (MTD) (square root of the product of length by breadth) was calculated. In the first experiment, mice in all groups were killed when the controls reached an MTD of 17 mm in accordance with UKCCCR Guidelines (1998). Primary tumours were excised, and snap-frozen in OCT (Leica, UK). In the second experiment, individual animals in each group were killed when their tumour reached an MTD of 17 mm.

### Immunohistochemistry

Sections (7 *μ*m) were cut from tissues preserved in OCT compound, fixed in cold acetone for 5 min, cold acetone: chloroform (1 : 1) for 5 min and cold acetone for 5 min followed by three washes in PBS for 5 min each.

Microvessel density was assessed as described previously (Harmey *et al*, 2002). In brief, following staining with an antibody to MECA32, an endothelial cell-specific marker, vessels were counted in five high power fields of view (h.p.f.) per section ( × 400 magnification, ( × 40 objective and × 10 ocular)) from five mice per group.

Apoptotic endothelial cells were identified with CD31 (to identify endothelial cells) and TUNEL (to identify apoptotic cells) staining. Sections fixed as above, were incubated with rat anti-mouse CD31 (BD-Pharmingen, San Jose, CA, USA) 1 : 400 in protein block (5%(v/v) normal horse serum, 1%(v/v) normal goat serum in PBS) overnight at 4°C, then washed three times in PBS. Sections were incubated with protein block for 10 min, then incubated with Texas Red conjugated goat anti-rat IgG (West Grove, PA, USA) diluted 1 : 200 in protein block for 90 min, and washed four times. Apoptosis was assayed using the DeadEnd Fluorometric TUNEL System (Promega, Madison, WI, USA) according to manufacturers instructions. Sections were then mounted using Prolong Gold Antifade Reagent with DAPI (Molecular Probes, Carlsbad, CA, USA). Apoptotic endothelial cells were counted in five high power fields of view (h.p.f.) per section ( × 400 magnification, ( × 40 objective and × 10 ocular)) from at least three mice per group.

Immunohistochemistry data were analysed by ANOVA with Bonferroni *post hoc* correction.

## Results

### IGF1R, IGFBP4 and PAPP-A expression by 4T1. 2 cells and 4T1.2 mammary fat pad tumours

**Figure 1:**
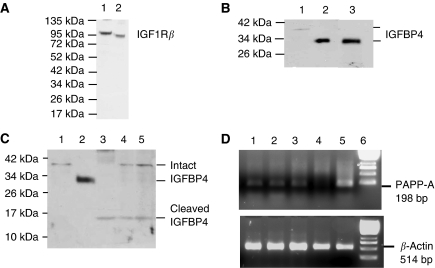
Expression of IGF1R, IGFBP4 and the IGFBP4 protease, PAPP-A. (**A**) Western blot analysis of IGF 1R*β* in 4T1.2 cell lysate (lane 1). Cell lysate from MCF7 cells is shown in lane 2 as positive control. Positions of molecular weight markers are indicated (kDa). (**B**) Western blot analysis of IGFBP4 expression in cell lysate (lane 1) and conditioned medium (lane 2) from 4T1.2 cells. Recombinant human IGFBP4 (10 ng) is shown in lane 3 as a positive control. Positions of molecular weight markers are indicated (kDa). (**C**) Western blot analysis of IGFBP4 in normal mammary fat pad (lane 3) and 4T1.2 mammary fat pad tumours (lanes 4 and 5). IGFBP4 expression in cell lysate (lane 1) and conditioned medium (lane 2) from 4T1.2 cells is shown for reference. Positions of molecular weight markers are indicated (kDa). (**D**) RT–PCR of PAPP-A. PAPP-A (top panel) was amplified by RT–PCR from cDNA prepared from the 4T1.2 mammary fat pad tumours (lanes 1, 2 and 3), 4T1.2 cells (lane 4) and MC3T3 mouse osteoblasts (lane 5). Molecular weight markers (1 Kb ladder) are shown in lane 6. *β*-Actin (lower panel) was amplified from all samples to control for sample quality.

4T1.2 cells express the IGF1R, identified by western blotting with an antibody specific for the IGF1R*β* subunit (Figure 1A). Cell lysates from MCF7 human breast cancer cells, which have previously been shown to express IGF1R, were used as a positive control (Hailey *et al*, 2002). The antibody used recognises both the mouse and human *β*-subunit of IGF1R. The predicted molecular weight for the human *β*-subunit is 95 kDa with the mouse subunit migrating slightly higher. 4T1.2 cells expressed IGFBP4, most of which is secreted (Figure 1B). Cellular IGFBP4 migrated slightly slower than the secreted IGFBP4, presumably because of the presence of the secretion signal. IGFBP4 expression in normal mammary fat pad tissue and 4T1.2 mammary fat pad tumours was then examined (Figure 1C). Cell lysate and conditioned medium from 4T1.2 cells was run alongside these samples to allow size alignment (lanes 1 and 2, respectively). Intact cell-associated IGFBP4 as well as IGFBP4 cleavage fragments were identified in both normal mammary fat pad tissue (lane 3) and 4T1.2 mammary tumours (lanes 4 and 5). PAPP-A cleaves IGFBP4 at a single site into two fragments of 18 and 14 kDa (Conover *et al*, 1995). The N-ter and C-ter cleavage fragments co-migrated as previously shown (Laursen *et al*, 2001). The presence of IGFBP4 cleavage fragments suggested the presence of active PAPP-A protease in these tissues. PAPP-A expression by 4T1.2 cells and in 4T1.2 mammary fat pad tumours was assessed by RT–PCR (Figure 1D). cDNA from differentiated mouse osteoblast MC3T3 cells previously shown to express PAPP-A was used as positive control (Bunn *et al*, 2004). Although 4T1.2 cells did not express PAPP-A, it was expressed within normal mammary fat pad tissue as well as 4T1.2 mammary fat pad tumours *in vivo*, suggesting that PAPP-A is produced by host cells within the tumours.

### Cell proliferation and VEGF expression in response to IGF1

**Figure 2:**
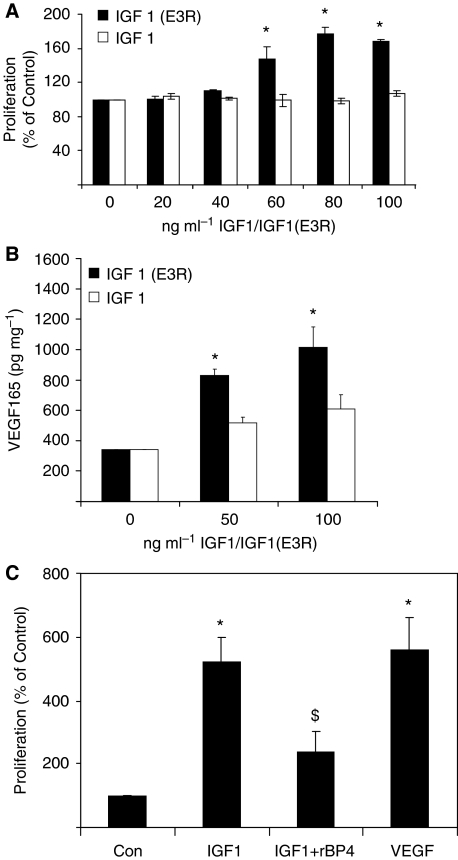
Effect of IGF1 on 4T1.2 tumour cells and MVECs. (**A**) 4T1.2 cell proliferation in response to IGF 1 or IGF 1 (E3R). Cells were treated in triplicate for 48 h and proliferation assayed by BrdU incorporation. Proliferation is expressed as % of control where control is taken as 100%. Data (*n*=3) expressed as mean±s.e.m. and analysed by ANOVA with LSD *post hoc* correction. ^*^P<0.05 *vs* Control. (**B**) VEGF expression by 4T1.2 cells in response to IGF 1 or IGF 1 (E3R). Cells were treated for 48 h in triplicate and VEGF_165_ measured by ELISA. Total cell protein was measured by BCA. Data (*n*=3) expressed as mean±s.e.m. and analysed by ANOVA with Scheffe *post hoc* correction. ^*^P<0.05 *vs* control. (**C**) Effect of IGFBP4 on IGF1-induced MVEC proliferation. Cells were treated in triplicate for 96 h with 50 ng ml^−1^ IGF1 alone or in combination with 200 ng ml^−1^ IGFBP4 and proliferation assessed by MTT assay. Cells were treated with 10 ng ml^−1^ recombinant VEGF as positive control. Proliferation is expressed as % of control where control is taken as 100%. Data (*n*=3) expressed as mean±s.e.m. and analysed by ANOVA with Tukey–Kramer *post hoc* correction. ^*^P<0.05 *vs* control; ^$^*P*<0.05 *vs* IGF1.

As 4T1.2 cells expressed IGF1R, cell proliferation in response to the IGF1 ligand was examined (Figure 2A). IGF1 had no effect on cell proliferation. However, IGF1 (E3R), a 70 amino-acid IGF1 analogue with substitution of Arg for Glu at position 3 with reduced affinity for all IGFBPs, significantly increased cell proliferation at doses ≥60 ng ml^−1^. The effect of biologically active IGF1 on the expression of the angiogenic factor, VEGF was then examined. IGF1 at doses ≥50 ng ml^−1^ had no significant effect on VEGF_165_ expression, while IGF1(E3R) significantly increased expression of VEGF_165_ at doses ≥50 ng ml^−1^ (Figure 2B). These data indicate that IGFBP4 (and/or other IGFBPs) secreted by 4T1.2 cells blocked IGF1 activity. As biologically active IGF1 increased VEGF production by 4T1.2 cells and has previously been shown to increase proliferation of primary HUVEC (human umbilical vein endothelial cells) (Wu *et al*, 2004), we examined the effect of IGF1 on proliferation of primary human dermal MVECs in the absence or presence of recombinant IGFBP4 (Figure 2C). IGF1 at 50 ng ml^−1^ increased MVEC proliferation, an effect that was blocked by 200 ng ml^−1^ rhIGFBP4.

### Protease-resistant IGFBP4 inhibits 4T1.2 tumour growth

**Figure 3:**
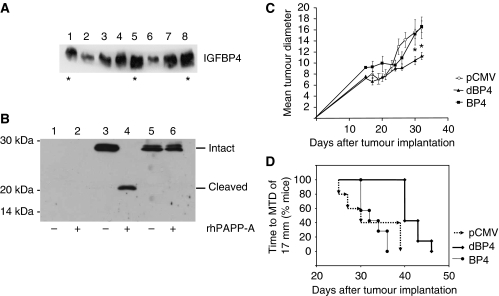
Protease-resistant IGFBP4 inhibits tumour growth. 4T1.2 tumour cells were transfected with control plasmid (pCMV), plasmid expressing wild-type IGFBP4 (pCMV-BP4) or plasmid expressing protease-resistant IGFBP4 (pCMV-dBP4). (**A**) Western blot showing IGFBP4 expression by control cells (lane 1) and single cell clones expressing dBP4 (lanes 2–5) or BP4 (lanes 6–8). Clones marked with an asterisk were used for *in vivo* studies. (**B**) HEK293T cells, which do not express endogenous IGFBP4, were transfected with pCMV (lanes 1+2), pCMV-BP4 (lanes 3+4) or pCMV-dBP4 (lanes 5+6). Conditioned medium was treated with rhPAPP-A in the presence of IGF2 for 24 h (−; untreated, +; PAPP-A-digested). Intact IGFBP4 and cleavage fragments were identified by western blot. (**C**) Tumour growth curve. 4T1.2 tumour cells transfected with control plasmid (pCMV), plasmid expressing wild-type IGFBP4 (pCMV-BP4) or plasmid expressing protease-resistant IGFBP4 (pCMV-dBP4) were implanted into the mammary fat pad of BALB/c mice (*n*=7 per group). Tumour diameter was measured on alternate days. Data (*n*=7) expressed as mean±s.e.m. and analysed by ANOVA with LSD *post hoc* correction. ^*^P<0.05 dBP4 *vs* pCMV or BP4. Data representative of two independent experiments. (**D**) Kaplan–Meier plot showing increased time to reach a mean tumour diameter (MTD) of 17 mm in mice with tumours expressing dBP4 relative to mice with tumours transfected with pCMV or BP4 (*χ*^2^=16.4, *P*<0.0001). Data representative of two independent experiments.

The *in vitro* data suggested that IGFBP4 would inhibit IGF1-stimulated growth of 4T1.2 tumours by blocking its effects on tumour cell and endothelial cell proliferation and VEGF production. *In vivo*, IGFBP4 is cleaved by the protease PAPP-A to release biologically active IGF1. We therefore examined the effects of wild-type IGFBP4 and protease-resistant IGFBP4 expression on tumour growth in an orthotopic model of breast cancer. Protease-resistant IGFBP4 and wild-type IGFBP4 were subcloned into pCMVScript under the control of the CMV constitutive promoter generating pCMV-dBP4 and pCMV-BP4, respectively. Figure 3A shows IGFBP4 expression by transfected 4T1.2 cells. The antibody used recognises both wild–type- and protease-resistant IGFBP4. Clones marked with an asterisk were used for *in vivo* studies. As we had no way of differentiating endogenous and exogenous IGFBP4 expression in 4T1.2 cells, pCMV, pCMV-BP4 and pCMV-dBP4 were transfected into HEK293 cells which do not express IGFBP4 (data not shown). Conditioned medium collected from these cells was treated with rhPAPP-A (500 ng ml^−1^) in the presence of IGF2 (10 *μ*g ml^−1^). Figure 3B shows that dBP4 is resistant to PAPP-A cleavage (lane 6), whereas the wild-type BP4 is completely cleaved (lane 4). The N-ter and C-ter cleavage fragments co-migrated as previously shown (Figure 1C and (Laursen *et al*, 2001)). The doublet band seen in the conditioned medium from dBP4-transfected HEK293 cells is probably a consequence of differential glycosylation as reported previously (Laursen *et al*, 2001).

4T1.2 cells transfected with pCMV, pCMV-BP4 or pCMV-dBP4 were implanted into the mammary fat pad of BALB/c mice. After 30 days tumours transfected with pCMV-dBP4 were significantly smaller (10.45±0.76 mm) than tumours transfected with pCMV-BP4 (15.26±0.87 mm), or pCMV (15.55±2.29 mm) (Figure 3C). In this experiment all mice were killed when tumours in the control group reached an MTD of 17 mm in accordance with UKCCCR guidelines (1998). In a second experiment, time to reach an MTD of 17 mm was used as a surrogate end point in accordance with UKCCCR guidelines (1998). Tumours transfected with pCMV-dBP4 took significantly longer to reach an MTD of 17 mm (41.7±2.2 days) than mice-bearing tumours transfected with pCMV (32±5.9 days) or pCMV-BP4 (32.6±2.5 days) (Figure 3D).

### Tumour angiogenesis

**Figure 4:**
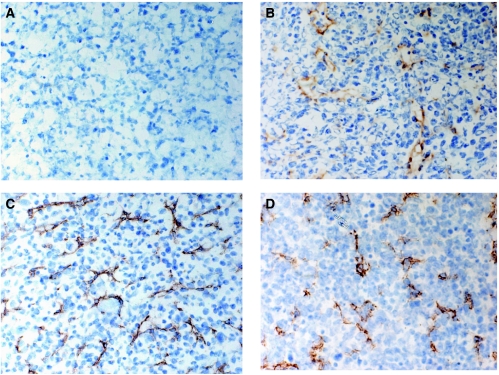
Angiogenesis in 4T1.2 mammary fat pad tumours. Sections were stained with anti-MECA32 to identify blood vessels. Negative control shown in (**A**). Representative sections stained for MECA32 from the mammary fat pad tumours transfected with (**B**) empty vector (pCMV), (**C**) plasmid expressing wild-type IGFBP4 (pCMV-BP4) or (**D**) plasmid expressing protease-resistant IGFBP4 (pCMV-dBP4). (Original magnification, × 400).

**Figure 5:**
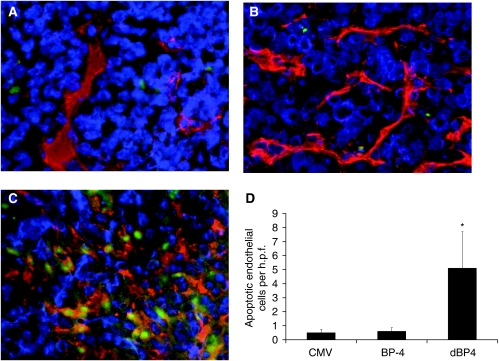
Protease-resistant IGFBP4 increases endothelial cell apoptosis. Sections of 4T1.2 mammary fat pad tumours transfected with (**A**) empty vector (pCMV), (**B**) plasmid expressing wild-type IGFBP4 (pCMV-BP4) or (**C**) plasmid expressing protease-resistant IGFBP4 (pCMV-dBP4) were stained for CD31 (to identify blood vessels), and TUNEL (to identify apoptotic cells). Red: CD31 positive, Green: TUNEL positive and Blue: nuclei stained with DAPI (original magnification × 400). (**D**) Apoptotic endothelial cells were counted in five high power fields of view (h.p.f.) per section from at least three mice per group. Data expressed as mean±s.e.m and analysed by ANOVA with Bonferroni *post hoc* correction. ^*^P<0.05 protease-resistant IGFBP4 (dBP4) *vs* empty vector or wild-type IGFBP4 (BP4).

Microvessel density in the mammary fat pad tumours transfected with pCMV (empty vector), wild-type IGFBP4 (BP4) or PAPP-A-resistant IGFBP4 (dBP4) was scored following MECA32 immunohistochemistry (Figure 4). Although there was no statistically significant difference in microvessel density between the different groups (*P*=NS, ANOVA with Bonferroni *post hoc* correction), the vessels within tumours expressing PAPP-A-resistant IGFBP4 (D), appeared to be of poor quality, with patchy staining suggesting a discontinuous endothelium and expanded cell cytoplasm; vessels were also occluded with no lumen visible, compared with tumours transfected with pCMV (B) or pCMV-BP4 (C). Endothelial cell apoptosis was then examined using a double stain for CD31 (to identify endothelial cells) and TUNEL (to identify apoptotic cells) (Figure 5). Tumours expressing PAPP-A-resistant IGFBP4 (C) had significantly higher numbers of apoptotic endothelial cells (6.97±3.26 s.e.m/h.p.f.) than tumours transfected with pCMV (0.90±0.50 s.e.m/h.p.f.) or pCMV-BP-4 (1.20±0.95 s.e.m./h.p.f.) (D).

## Discussion

*In vivo* the activity of IGF is regulated by the IGF-binding proteins, which function to either enhance or inhibit the mitogenic activities of IGF1 (Jones and Clemmons, 1995). The majority of IGF is synthesised within the liver and transported in the bloodstream bound to the IGFBPs. When bound to IGFBP4, IGF1 is biologically inactive but IGFBP4 can be cleaved by the IGFBP4-specific protease, PAPP-A, to release active IGF1 (Conover *et al*, 2004).

4T1.2 cells expressed the IGF1R and IGFBP4, but not the IGFBP4 protease PAPP-A, although PAPP-A was expressed within 4T1.2 mammary fat pad tumours. IGFBP4 cleavage fragments were identified in 4T1.2 mammary fat pad tumour tissue but not in cultured 4T1.2 cells or conditioned medium, suggesting that PAPP-A within the tumours was produced by host cells. Within the tumour environment, it is likely that the secretion of IGFBP4 in the presence of the IGFBP4 protease (produced by host cells, such as fibroblasts and osteoblasts) provides a steady supply of IGF1 to the tumour to support its growth and angiogenesis. IGF1(E3R), a recombinant form of IGF1, which is resistant to IGFBP binding and therefore retains its activity even in the presence of IGFBPs, increased 4T1.2 cell proliferation and production of the angiogenic factor, VEGF. Wild-type IGF1 did not increase 4T1.2 cell proliferation and had no effect on VEGF expression suggesting that the IGF1 is bound by IGFBP4 (and/or other IGFBPs) secreted by the 4T1.2 cells and rendered biologically inactive, while IGF1(E3R) remains unbound and thus biologically active. In addition to its effects on 4T1.2 tumour cells, IGF1 stimulated growth of human dermal MVECs, an effect that was blocked by recombinant IGFBP4. These data indicated that IGF1 would promote angiogenesis and that IGFBP4 could block IGF1-stimulated angiogenesis.

We then examined the effect of IGF1 blockade on growth of 4T1.2 mammary tumours *in vivo*. The approach we used was to transfect 4T1.2 cells with a protease-resistant IGFBP4. The 4T1.2 orthotopic model used here arose from a spontaneous mammary tumour in a BALB/c mouse and spontaneously metastasises to lung and bone recapitulating breast tumour tissue microenvironment and cell–cell interactions (Lelekakis *et al*, 1999). It is therefore a clinically relevant model, which reflects the behaviour of human breast cancer. The protease-resistant IGFBP4 clone used (dBP4) was previously shown to be resistant to cleavage by a smooth muscle cell-derived protease, but its binding affinity for IGF1 was equivalent to wild-type IGFBP4 (Zhang *et al*, 2002). Previous studies had demonstrated that this dBP4 construct was resistant to cleavage by a protease present in conditioned medium from smooth muscle cells, but did not unequivocally show that the protease was PAPP-A (Zhang *et al*, 2002). We demonstrated that this construct was resistant to cleavage by recombinant PAPP-A. IGF will therefore remain bound to this IGFBP4 and inactive even in the presence of PAPP-A. 4T1.2 cells transfected with pCMV (empty vector), pCMV-BP4 (plasmid expressing wild-type IGFBP4) or pCMV-dBP4 (plasmid expressing protease-resistant IGFBP4) were implanted into the mammary fat pad of BALB/c mice. Tumours expressing protease-resistant IGFBP4 grew significantly more slowly than tumours transfected with pCMV or pCMV-BP4 (measured as time to reach an MTD of 17 mm). As IGF1 increased VEGF production by the 4T1.2 cells and stimulated MVEC proliferation, we anticipated that an antiangiogenic effect might underlie this inhibition of tumour growth. We therefore examined microvessel density and morphology within the tumours by MECA32 staining. Although there was no statistically significant difference in vessel number in the tumours of all three groups at the time point examined, vessels in tumours expressing PAPP-A-resistant IGFBP4 were of poorer quality than in the other groups with patchy staining and occluded lumen. A similar effect was previously observed in tumours treated with the angiogenesis inhibitor, alphastatin, where vessels also had a discontinuous endothelial cell wall and occluded lumen (Staton *et al*, 2004). Tumour sections were then stained with CD31 and TUNEL to identify apoptotic endothelial cells. There were almost no apoptotic endothelial cells in control tumours or those expressing wild-type IGFBP4, but there were large numbers of apoptotic endothelial cells in the tumours expressing protease-resistant IGFBP4. Our data clearly show that expression of PAPP-A-resistant IGFBP4 results in endothelial cell apoptosis within the tumours and inhibition of tumour growth. Although we saw increased endothelial cell apoptosis in tumours expressing protease-resistant IGFBP4, this was not reflected in reduced microvessel density. This was probably because at the time point analysed non-transfected cells would likely predominate within the tumour with a concomitant decrease in dBP4 expression and regrowth of vessels.

In contrast to our findings in an orthotopic model of breast cancer where wild-type IGFBP4 had no effect on tumour growth, wild-type IGFBP4 inhibited tumour growth in non-orthotopic murine models of colon cancer and prostate cancer where transfected cells were implanted subcutaneously (Damon *et al*, 1998; Durai *et al*, 2007). However, subcutaneous expression of the PAPP-A protease may be quite different to that in either the colon or the prostate gland. Our data show that PAPP-A is present and active in both normal mammary tissue and 4T1.2 mammary fat pad tumours where the PAPP-A is produced by host cells within the tumours. Therefore, in the mammary tissue at least, the interaction between the tumour cells and associated host cells is important for PAPP-A-mediated cleavage of IGFBP4 and therefore control of IGF bioavailability and activity. The importance of using orthotopic mouse models in cancer studies has been highlighted recently (Fidler, 2006). Tumour microenvironment plays a critical role in tumour growth, particularly angiogenesis and metastasis, both of which are regulated by host factors as well as tumour factors (Fidler, 2003). As PAPP-A is expressed by fibroblasts and osteoblasts, host cells in and around tumours are likely to play an important role in proteolysis of IGFBP4 (Conover *et al*, 1993; Durham *et al*, 1995).

Wild-type IGFBP4 may paradoxically increase tumour growth by serving as a reservoir of IGF1. Indeed, systemic administration of wild-type IGFBP4 increased bone formation parameters (osteocalcin, alkaline phosphate) in mice by increasing IGF bioavailability (Miyakoshi *et al*, 2001). The approach described here – PAPP-A-resistant IGFBP4 – will inhibit IGF activity even in the presence of the PAPP-A protease and there is some evidence that PAPP-A plays a role in human breast cancer progression (Kuhajda *et al*, 1985).

There are a number of strategies to block IGF under investigation, some of which are in clinical trials. An antibody, *α*IR3, directed against the IGF receptor, IGF1R, blocked IGF activity and tumour growth in xenograft models of human breast cancer, but the antibody had some agonist activity and cross-reactivity with the insulin receptor (Kato *et al*, 1993). Insulin-like growth factor antagonists which act by blockade of interactions at the IGF1R may also significantly alter insulin action at the insulin receptor, a serious and unacceptable side effect. As multiple IGF receptors exist, targeting IGF itself is likely to be more effective than targeting IGF1R.

The IGFBPs are specific for the IGFs and should not affect insulin signalling. IGFBP2 and IGFBP3 have been examined as approaches to block IGF signalling. As the IGFBPs bind IGF with higher affinity than IGFR1, they should effectively compete with the receptor for IGF ligand binding (Clemmons, 1997). rhIGFBP3 (recombinant human IGFBP3) was shown to potentiate the activity of Herceptin against MCF7 xenografts engineered to overexpress HER2 (Jerome *et al*, 2006). Unlike IGFBP4, which is always inhibitory, other IGFBPs can either inhibit or potentiate IGF activity depending on the context and have IGF-independent effects as well as IGF-dependent activity. Under certain conditions IGFBP3 increased IGF-dependent cell survival and proliferation, possibly by enhanced delivery of IGF1 to IGF1R (De Mellow and Baxter, 1988). IGFBP3 acts as the major carrier of IGF1 in the circulation where it exists as a complex with the ALS (acid labile subunit). This complex acts as a reservoir of IGF1, which can be mobilised by proteolysis of IGFBP3. This complex does not cross the endothelial barrier and complex formation with ALS may impair delivery of rIGFBP3 to tumour tissue (Payet *et al*, 2004). We believe that our approach using a protease-resistant IGFBP4 to block IGF activity is superior to those described above, as it inactivates IGF1, is small enough (24–32 kDa) to cross the endothelial barrier and should have a long half-life *in vivo* due its resistance to the PAPP-A protease. In addition to the data presented here, a protease-resistant IGFBP4 was recently shown to inhibit IGF and therefore neointimal expansion in a model of neointimal hyperplasia (Nichols *et al*, 2007). It is a relatively small molecule compared with antibody therapies, and hence may penetrate tumours more effectively. The data presented here showing that a protease-resistant IGFBP4 blocks IGF activity and therefore tumour growth and angiogenesis, suggests that protease-resistant IGFBP4 may have value as a novel breast cancer treatment. We are currently investigating the therapeutic potential of recombinant protease-resistant IGFBP4 in breast cancer.
